# Transplanting Neural Progenitor Cells Improves Neural Regulation But Not Hormonal Reliance of Cardiovascular Function Following Spinal Cord Injury

**DOI:** 10.1523/ENEURO.0030-25.2025

**Published:** 2025-08-29

**Authors:** Cameron T. Trueblood, Fateme Khodadadi-Mericle, Zhifeng Qi, Silvia Fernandes, Theresa Connors, Micaela L. O’Reilly, John D. Houle, Veronica J. Tom, Shaoping Hou

**Affiliations:** ^1^Marion Murray Spinal Cord Research Center, Department of Neurobiology and Anatomy, Drexel University College of Medicine, Philadelphia, Pennsylvania 19129; ^2^Dalton Cardiovascular Research Center, Department of Pathology & Anatomical Sciences, Department of Physical Medicine & Rehabilitation, University of Missouri School of Medicine, Columbia, Missouri 65211; ^3^Harry S. Truman Memorial Veteran Hospital, Columbia, Missouri 65201

**Keywords:** autonomic dysreflexia, cell transplantation, hemodynamics, renin–angiotensin system, serotonin

## Abstract

High-level spinal cord injury (SCI) often reduces neural regulation of cardiovascular function. During the chronic phase, humoral regulation via the renin–angiotensin system (RAS) is enhanced to compensatorily maintaining blood pressure. It was recently shown that transplanting early-stage neurons into the injured cord mitigates cardiovascular disorders. However, the mechanisms underlying this recovery remain largely unknown. Here, we employed various pharmacological interventions to elucidate whether this strategic transplantation affects the imbalance of neuroendocrine regulation of hemodynamics and the role of specific serotonergic and catecholaminergic components. Female rats received a complete crush at the fourth thoracic spinal cord. Embryonic neural progenitor cells (NPCs) harvested from the raphe nuclei or the spinal cord were transplanted into the lesion. Naive rats or injury alone served as controls. After 8–9 weeks, radio-telemetric recordings demonstrated that both implants decreased tachycardia at rest and diminished the frequency or severity of autonomic dysreflexia (AD). Pharmacological interventions demonstrated that both NPC grafts partially restored neural regulation of blood pressure without normalizing the aberrant RAS hyperactivity. Subsequently, specific neural mechanisms were explored through intrathecal administration of the 5-HT_2A_ antagonist ketanserin, the 5-HT_1A_ antagonist WAY100635, or the α1-adrenoreceptor antagonist prazosin. It revealed that graft-derived serotonergic signaling was involved in the restoration of the resting heart rate via 5-HT_2A_ receptors but did not attenuate AD. In addition, catecholaminergic mechanisms remained critical for blood pressure maintenance after SCI. Ultimately, the results provide insight into understanding the mechanistic nuances associated with cell therapy for SCI-induced cardiovascular dysfunction.

## Significance Statement

High-level spinal cord injury (SCI) often impairs neural regulation of cardiovascular function, inducing compensatory hyperactivation of the renin–angiotensin system (RAS) to maintain blood pressure. Transplanting embryonic neural progenitor cells, harvested from the fetal raphe nuclei or spinal cord, partially restored neural control of the vasculature but did not ameliorate high RAS reliance. Graft-derived serotonin transmission was involved in the restoration of hemodynamics via 5-HT_2A_ receptors but not dysreflexic responses. Meanwhile, catecholaminergic mechanisms continued to mediate blood pressure regulation after SCI. The results provide new insight to better understand the mechanistic nuances associated with cell therapy for SCI-induced cardiovascular dysfunction.

## Introduction

Traumatic spinal cord injury (SCI) at high levels often interrupts supraspinal vasomotor pathways and results in cardiovascular dysfunction. The loss of cerebral regulation of sympathetic activity renders unstable hemodynamics at rest, orthostatic hypotension, and the development of autonomic dysreflexia (AD) in the chronic phase (Krassioukov et al., 2002; Eldahan and Rabchevsky, 2018). As cardiovascular dysfunction is a leading cause of morbidity and mortality in patients with SCI, it is highly important to investigate the underpinnings of this disorder and explore the efficacy of potential therapeutics. Previously, transplanting early-stage neurons into the injured spinal cord was shown to provide a substrate to reconnect the damaged neuronal pathways and relay signal transduction from higher centers to the caudal spinal cord, thereby partially restoring cardiovascular function (Hou et al., 2013b, 2020).

Although cell transplantation can improve cardiovascular regulation after SCI, the precise mechanisms underlying these functional improvements are largely unknown. Under normal conditions, the autonomic nervous system (ANS) is a primary regulator of the cardiovascular system. However, hemodynamic function is also regulated through activation of the renin–angiotensin system (RAS; Allen et al., 1988; Bilodeau and Leiter, 2018). Unlike the ANS, which maintains acute cardiovascular homeostasis, the RAS-mediated regulation is typically responsible for long-term changes in blood pressure and fluid volume (Burns et al., 1993). After SCI, neural regulatory mechanism is disrupted, resulting in chronically low resting blood pressure. In response to the change, renin is released from the kidneys which in turn triggers the production of angiotensin II, a vasoconstricting hormone, into the bloodstream and various tissues (Davis and Freeman, 1976; Fountain and Lappin, 2021). It has recently been demonstrated that RAS regulation of cardiovascular function may be augmented after an SCI interrupts descending control of the sympathetic drive (Lujan et al., 2018), suggesting that neuroendocrine control is out of balance.

Transplanting neural progenitor cells (NPCs) into the spinal cord lesion can both compensate for injured neuronal pathways and serve as a source of specific neurotransmitters in the spinal cord caudal to the injury. Earlier investigations reported the potential for cell transplantation to improve somatic and autonomic functions (Jakeman and Reier, 1991; Lu et al., 2012; Hou et al., 2013b). More recently, transplantation of NPC, harvested from embryonic rat raphe nuclei, was found to enhance serotonergic regulation of cardiovascular function (Hou et al., 2020). Though the formation of a graft-derived relay between supraspinal vasomotor centers and spinal sympathetic preganglionic neurons (SPNs) suggests partial restoration of sympathetic regulation, the degree to which transplanted cells mediate functional improvements remains to be determined.

While complete spinal cord transection rat models have been used to study cell transplantation therapeutics, they are suboptimal due to poor graft–host integration. It was previously found that crush spinal cord injuries demonstrate less fibrotic scarring and cavitation within the lesion site compared with complete spinal cord transection, creating a more permissive environment for cell survival and integration (Hou et al., 2018). Using this optimized transplantation system (Trueblood et al., 2019), in the present study, we examined the mechanisms underlying NPC graft-derived cardiovascular functional recovery. The findings may guide cell selection for future clinical trials.

## Materials and Methods

### Animals

**Figure 1.**
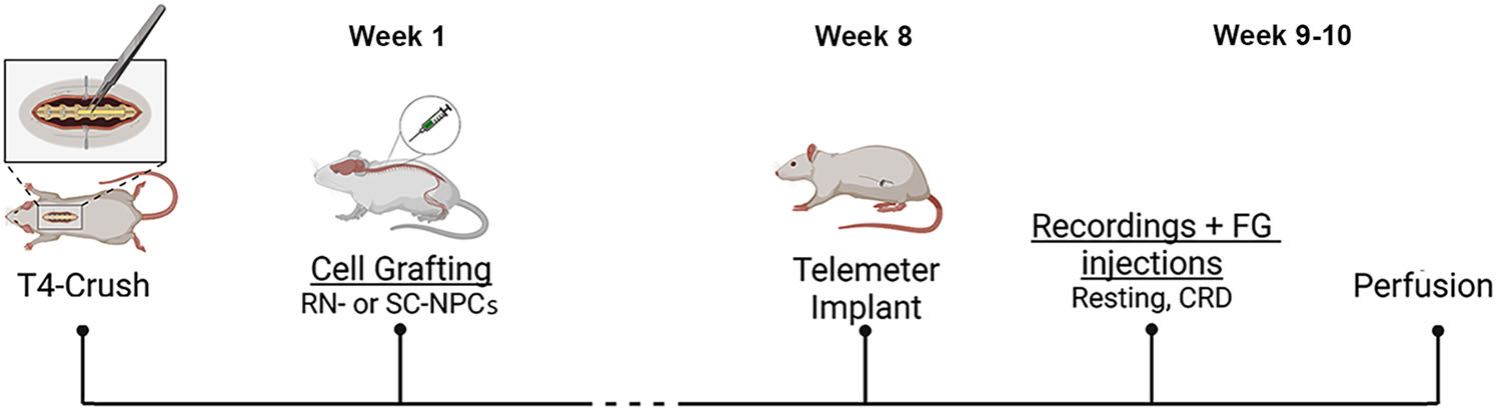
A timeline for the experimental procedure. Created with Biorender.com.

Adult female Fisher 344 rats (*n* = 121; 150–175 g; 3–4 months, Charles River Laboratories) were used for experiments. All rats were cared for according to Drexel University and the University of Missouri's Institutional Animal Care and Committee guidelines throughout experimental procedures. The animals had unlimited access to food and water and were housed three per microisolator cage. All cages were maintained in a temperature and light-controlled environment. Animals were allowed a 1 week acclimation period upon arrival before any subsequent experimentation. In cell transplantation study, the in vivo procedures were ∼9–10 weeks including SCI, cell grafting, and cardiovascular parameter analysis (Fig. 1). The rats were randomly divided into four cohorts: (1) naive controls (*n* = 19), (2) T4-crush injury (*n* = 46), (3) T4-crush with NPC harvested from the raphe nucleus (RN-NPC) grafting (*n* = 20), and (4) T4-crush with NPC harvested from the spinal cord (SC-NPC) grafting (*n* = 23). To further validate cardiovascular consequences of this spinal cord crush model, an additional group of rats underwent complete T4 spinal cord transection (*n* = 13), which is a well-established injury model for hemodynamic disorders, for comparison as a pilot study. After all surgical procedures, rats received cefazolin (10 mg/kg), buprenex (0.035 mg/kg) for pain management, as well as lactated Ringer's for recovery. Animals with SCI were placed on thermal pads for the duration of the study and received manual bladder expression three times daily for the first 2 weeks postinjury and then twice daily until killed.

### SCI

All animals were anesthetized through inhalation of 4% isoflurane for surgical preparation followed by 2% isoflurane for sustainment. An incision of the skin (2–3 cm) was created in the skin of the back directly caudal to the T2 spinal process. The T3 spinal process was then exposed by separating the overlying connective tissue and muscle using a tissue separator. After a T3 dorsal laminectomy, the T4 spinal cord was completely crushed with fine forceps (*n* = 89) or transected using a surgical #11 blade (*n* = 13). To crush the spinal cord, the forceps tips were positioned laterally on either side of the cord, touching the ventral vertebrae and pressed tightly together for 10 s. Visual verification of injury was used to ensure that a crush lesion was induced. Due to the intact dura, the lesion site was narrow without spinal cord retraction. For spinal cord transection, injury completeness was initially assessed via visual verification of spinal cord stump retraction regarding the opened dura. Once all active bleeding was controlled, the muscles were sutured, and the skin was subsequently stapled. As an exclusion criterion and simple indices of hindlimb motor function, the Basso, Beattie, and Bresnahan testing was implemented in the first 3 weeks after SCI alone, either crush or transection, and injury followed by cell transplantation described below. If a score is >3, it often indicates that there was spared tissue mediating residual function, and the rat was excluded from further experiments (Hou et al., 2018; Trueblood et al., 2019). The completeness of spinal cord lesion was further verified in a series of spinal cord sections by histological analysis after perfusion, and rats with incomplete lesions were also excluded (Hou et al., 2018).

### Cell preparation and transplantation

Serotonergic populations of the caudal raphe, including the raphe magnus, obscurus, and pallidus, project directly to the spinal cord and play a critical role in the maintenance of hemodynamic function (Loewy and McKellar, 1981; Minson et al., 2002; Hornung, 2003). In this study, we transplanted embryonic cells harvested from the raphe region of the brainstem to enrich the serotonergic population, while transplanted cells collected from the spinal cord have no or very few serotonergic neurons. The local inflammatory reaction responsible for the injury is detrimental to grafted cells. Instead of immediate transplanting, delayed grafting can avoid the peak of inflammation and decrease the rate of cell death (Hou et al., 2020). To achieve maximum cell survival, the spinal cord lesion was re-exposed 1 week post-SCI for cell transplantation. RN- or SC-NPCs were dissected and disassociated from Day 14 (E14) transgenic embryos ubiquitously expressing green fluorescent protein (GFP; Rat Resource and Research Center, University of Missouri). The brainstem was mechanically dissected, and the tissue was opened and placed in a flat position (Hou et al., 2020). Regarding the raphe cells in the middle of the tissue, the region of ∼0.5 mm from the edge was cut and removed. The mid part, including raphe nuclei, was harvested and subsequently chemically dissociated according to the method described by Harris et al. (2007). This method did not purify serotonergic cells, and other types of neurons and glial cells were included in the grafts, which was beneficial for survival. We previously confirmed that this increased the percentage of serotonergic neurons to ∼12% to neuronal cells (Hou et al., 2020). The spinal cord tissue was collected and dissociated as well. Prior to transplantation, the RN or SC cell solution was resuspended into a fibrin matrix (25 mg/ml fibrogen, 25 U/ml thrombin), respectively, containing the following growth factors for supplementary support graft survival as previously described (Lu et al., 2012; Hou et al., 2013b): BDNF (50 µg/ml), platelet-derived growth factor-AA (10 µg/ml), neurotrophin-3 (50 µg/ml), basic fibroblast growth factor (10 µg/ml), glial cell line-derived neurotrophic factor (10 µg/ml), and hepatocyte growth factor (10 µg/ml). Cellular samples were then placed immediately on ice in preparation for transplantation.

One week following injury, animals were anesthetized with isoflurane, and the spinal cord lesion was re-exposed. Resuspended RN-NPCs and SC-NPCs were injected into the lesion site of the crushed spinal cord using a 5 µl Hamilton syringe connected with a glass tip, respectively. A total of 8 µl of either E14 RN- or SC-NPCs (1.0 × 10^6^/µl) were microinjected into the lesion. The dura remained intact allowing for maximal retention of grafted cells during the procedure. The overlaying muscle and skin were then sutured and stapled, respectively. Animals received injections of antibiotics and analgesia drugs for postsurgical care as described before.

### Radio-telemeter implantation

To monitor and compare hemodynamic parameters before and after SCI, animals for injury model validation at the acute/subacute phase were implanted with telemeters 1 week prior to injury. In the model validation at the chronic phase and cell transplantation as well as control groups, telemeter probes were implanted 8 weeks posttransplantation to record blood pressure and the heart rate (HR). A radio-telemeter (model HD-S10, Data Sciences International) was implanted into the abdominal cavity for hemodynamic recordings. The rats were deeply anesthetized through inhalation of 4% isoflurane for surgical prep, followed by 2% isoflurane for sustainment during surgery. A skin incision was made at the midline of the ventral abdomen, and the underlying muscle layer was incised to expose the abdominal cavity. The organs were then gently moved aside, and the descending aorta was separated from the vein. After securing the vessel between the rostral and caudal bifurcation sites, the catheter of the radio-telemetric pressure transducer was tipped rostrally into the abdominal descending aorta for 3–4 cm. A drop of tissue glue was applied to seal the lesion. Subsequently, the transducer body was anchored to the abdominal muscle wall. Once the viscera were repositioned to their original location, the muscle wall and skin were then sutured, respectively. The implanted telemeter allowed for real-time analysis of mean arterial pressure (MAP) and HR parameters.

### Hemodynamic recordings

The Ponemah software (Data Sciences International) was employed for telemetric monitoring of hemodynamics. With this program, real-time pulse arterial pressure was transfigured as a radiofrequency signal and transmitted to a receiver corresponding to an individual rat. The data were then exported and analyzed. For one subset of rats (*n* = 28), hemodynamic parameters were recorded preinjury, 1 d (1DPI), and 2 week postinjury (2WPI). All other SCI rats (*n* = 76) underwent cardiovascular recordings 9 weeks postinjury, while hemodynamics in naive controls (*n* = 19) were obtained 1 week after telemeter implantation.

#### Resting MAP and HR

Hemodynamic parameters were recorded at rest. Singly housed rats were placed on receivers associated with their telemeters. Once the telemeters were turned on, rats were left undisturbed for a 10–15 min acclimation period to allow for stabilization of parameters. Basal MAP and HR were then recorded for a total of 15 min with a sampling rate of 10 s. To minimize interference from environmental stressors, the testing room was kept in a quiescent state throughout the experiment. The recording was performed at a certain time (10:00–10:15 A.M.) per day for all animals to avoid circadian differences. Afterward, the collected MAP and HR values per animal were averaged for statistical analysis.

#### Events of spontaneous AD

The number of spontaneously occurring AD events was measured over 24 h period. MAP and HR parameters were recorded using the Ponemah software and subsequently analyzed with a specific algorithm script written in MATLAB (Mironets et al., 2018). The algorithm assessed the number of instances in which a substantial increase in MAP (>20 mmHg) was accompanied by extensive bradycardia, signified by a decrease of at least 20 beats per minute (bpm). Only episodes that persisted longer than 30 s were identified as AD events. *Ad libitum* moving rats were situated and prepared for this procedure as previously described. Prior to recordings, the animals had their bladders expressed to ensure all subjects had a similar baseline for AD induction. Cardiovascular parameters were recorded at a sampling rate of 2 s for 24 h. The average number of AD events over two 24 h time periods was calculated for statistical analysis.

#### Colorectal distention (CRD)-induced AD

To examine the severity of an AD episode, we performed CRD to artificially induce AD. A balloon-tipped catheter coated with a petroleum-based lubricant was inserted 2 cm into the rectum. The catheter was then secured to the rat's tail using a tape. Once anchored, the rats were gently restrained in a cloth towel to create a calming environment. To induce an AD episode, we then inflated the balloon with 1.4 ml of air using an attached syringe and remained inflated for 1 min (Maiorov et al., 1997; Hou et al., 2013a). This procedure expands the bowel in a similar fashion to fecal bowel impaction. MAP and HR parameters were recorded 1 min prior to, during, and after distention at a sampling rate of 3 s. Each rat underwent two trials of this procedure with a 15 min rest period between adjacent recordings to allow cardiovascular parameters to return to the baseline. Upon completion, the rats were given buprenex subcutaneously for pain relief. The differences in changes in MAP and HR for each animal were calculated and subsequently averaged across the two trials.

### Systemic blockade of neural/hormonal regulation for hemodynamics

Cardiovascular function is regulated through not only the ANS but also the RAS. It was recently reported that RAS regulation of cardiovascular function is augmented after an SCI disrupts descending control of sympathetic drive (Lujan et al., 2018). To investigate the possible dynamic change of neural (mainly sympathetic) and hormonal domination of hemodynamic function after injury/cell transplantation, we administered the ganglionic blocker hexamethonium chloride (Hexa) and the angiotensin-converting enzyme (ACE) inhibitor captopril, respectively (Table 1).

**Table 1.** Drug information

Drug	Activity	Route	Dose	
			Resting BP/HR	CRD
Hexa	Ganglionic blocker	Intraperitoneal	30 mg/kg	
Captopril	ACE inhibitor	Intraperitoneal	1 mg/kg	
Ketanserin	5-HT_2A_ antagonist	Intrathecal	5 µg/kg	20 µg/kg
			20 µg/kg	
WAY 100635	5-HT_1A_ antagonist	Intrathecal	10 µg/kg	50 µg/kg
			50 µg/kg	
Prazosin	α1 antagonist	Intrathecal	1 µg/kg	1 µg/kg

Although sympathetic and hormonal regulation of cardiovascular function has been thought to work through distinct mechanisms, recent work has demonstrated that presympathetic nuclei are directly influenced by the hormone angiotensin (Kumagai et al., 2012; Bilodeau and Leiter, 2018). Angiotensin regulation of resting hemodynamics largely acts directly on the vasculature to induce vasoconstriction. It has been found that the RAS pathway also acts centrally through manipulation of the sympathetic nervous system (Reid, 1992). Previous studies have shown that captopril effectively inhibits the production of the vasoconstriction hormone, angiotensin II (Hanif et al., 2010). This hormone, in turn, has been reported to influence supraspinal regulators of sympathetic activity (Wang et al., 2007, 2017). Accordingly, we utilized a dual administration approach to determine the pure neural and hormonal contribution to hemodynamic activity in RN-NPC–grafted rats. Vehicle injections served as controls. This approach would reveal pure machinery of neural or hormonal regulation of hemodynamics.

#### Single drug administration

To validate the crush model's response similar to transection, one cohort of rats received the drug at preinjury and 2WPI. A total bolus of 200 µl of each drug was administered intraperitoneally (Hexa 30 mg/kg, captopril 1 mg/kg). A 15 min rest period was given to allow the drug to diffuse systemically. Subsequently, resting MAP and HR were continuously recorded for 15 min. Parameters were measured as percentage change from the baseline after drug administration.

#### Dual pharmacological administration

In one cohort of animals, including the naive and injury with or without NPC grafting, Hexa (30 mg/kg) or captopril (1 mg/kg) was administered intraperitoneally after initial equilibration. Fifteen minutes later, resting MAP and HR parameters were recorded for 15 min, as described above. At the end of the first recordings, while the primary drug was still in effect, the second drug was administered, respectively. After allowing 15 min for diffusion, resting MAP and HR were recorded for a final 15 min period. A total volume of 200 µl of each drug was administered intraperitoneally. We calculated the difference in MAP and HR between the dual administration response and the primary drug response. With this approach, we were able to examine neural output after SCI without the angiotensin influence and determine if NPC grafting affects neural regulation of cardiovascular function.

### Centrally pharmacological interventions of neural receptors for cardiovascular function

To investigate if RN-NPC grafting restores serotonergic regulation of hemodynamic function, we employed pharmacological reagents to manipulate intraspinal serotonergic receptors, including the 5-HT_2A_ antagonist ketanserin and the 5-HT_1A_ antagonist WAY 100635. Our preliminary results indicated that a population of catecholaminergic neurons in the NPC graft might exert effects on cardiovascular recovery. Accordingly, the α_1_-adrenoreceptor antagonist prazosin was also delivered to determine its involvement (Table 1).

#### Central 5-HT receptor blockade

The rats were anesthetized as previously described and underwent an L5 laminectomy to gain access to the intrathecal space. Subsequently, a small-diameter hole was created in the dura, and a catheter was inserted 2–3 cm rostrally. The most proximal portion of the catheter was then sutured in place between the surrounding musculature and skin, leaving 1–2 in of the outermost portion exposed. The rats were then placed on their respective receivers and given at least a 30 min period to recover from the anesthesia. All experimental pharmacological agents were injected with a volume of 10 µl, 10–15 s prior to resting hemodynamic recordings in increasing concentrations. Ketanserin was given in dosages of 5 and 20 µg/kg, whereas WAY 100635 was administered at 10 and 50 µg/kg. Directly following drug inoculation, 5 µl of saline (vehicle) was injected to ensure full delivery of the drug from the catheter. Thereafter, resting MAP and HR parameters were recorded for 15 min for analysis. Between different drug administrations, a 30 min period of elapse was allowed to ensure drug washout. To examine pharmacological effects on AD, we employed the CRD as previously described. Rats received only the high dose of the drugs 10–15 s prior to CRD induction, with a 30- in washout period between each drug. The animals were subjected to two trials of CRD with a 15 min rest period between each recording. Buprenex was subsequently administered for pain relief at the completion of CRD experiments.

#### Central adrenergic receptor blockade

The effects of epinephrine/norepinephrine on resting hemodynamics and CRD-induced AD response were examined by intrathecally administering the α_1_-adrenoreceptor antagonist prazosin (1 µg/kg). The administration protocol matched that described above.

### Fluorogold injection to label SPNs

One week before killing, one cohort of animals for histological analysis, including RN-NPC grafting (*n* = 5), SC-NPC grafting (*n* = 4), and injury-only rats (*n* = 4), received intraperitoneal injection of Fluorogold (FG; 0.2 ml of 0.5% in distilled water; fluorochrome) to retrogradely label SPNs within the intermediolateral (IML) zone of the spinal cord (Akhavan et al., 2006).

### Histological analysis

#### Tissue processing

After being overdosed with intraperitoneal injections of Euthasol, animals were transcardially perfused with ice-cold 0.9% saline and then 4% paraformaldehyde. The spinal cord was then dissected and incubated in 4% paraformaldehyde overnight for postfixation. Afterward, the tissue was placed in 30% sucrose for at least 24 h. A 3 cm spinal cord segment containing the lesion/transplantation site (0.5 cm rostral and 2.5 cm caudal) was subsequently embedded in a tragacanth medium (Millipore Sigma) and frozen for cryosectioning. The spinal cord was then serially sectioned at 35 µm into horizontal longitudinal slices in six series of free-floating sections (Hou et al., 2013a).

#### Immunostaining

To investigate graft integration across the lesion site, we performed triple immunostaining for GFP, glial fibrillary acidic protein (GFAP), and platelet-derived growth factor receptor β (PDGFR-β). Though gliotic scarring has been considered a negative factor for central nervous system axon regeneration in earlier SCI studies, recent investigations have challenged this opinion and demonstrated that astrocyte scar formation aids rather than inhibits axon regeneration, mainly via the expression of multiple axon-growth–supporting molecules (Anderson et al., 2016). In cell transplantation studies, we previously identified that astrocyte gliosis is not an issue for grafted cell survival and axon growth. In contrast, fibrotic scarring, which can be disclosed with PDGFR-β labeling, generates a physical barrier for graft–host integration (Hou et al., 2018, 2020). Accordingly, this triple staining could evaluate PDGFR-β–labeled fibrotic scarring between GFP^+^ grafts and host tissue identified by GFAP labeling. Since nearly all grafted spinal cords lacked PDGFR-β–labeled fibrotic scarring along the edge of the graft, a qualitative description but not quantification was implemented for the evaluation. The 5-HT and dopamine beta-hydroxylase (DBH) antibodies were used to identify graft-derived serotonergic and noradrenergic cell bodies and axonal projections, respectively. Spinal sections were submerged in Tris buffer solution (TBS) for washing followed by incubation in 30% hydrogen peroxide for 15 min. Subsequently, the sections were submerged in TBS once more prior to being blocked in TBS containing 0.5% Triton X-100 and 5% donkey serum for 1 h. The blocked tissue was then incubated with distinct combinations of primary antibodies, including goat anti-GFAP (1:1,000; Abcam), rabbit anti-PDGFR-β (1:1,000, Abcam), mouse anti-Collagen III (1:1,000, Abcam), chicken IgY anti-GFP (1:500, Abcam), rabbit anti-5-HT (1:1,000, Immunostar), and mouse anti-DBH (1: 500, Abcam) overnight at 4°C. On the second day, following three washes in TBS, the tissue was placed in an appropriate Alexa Fluor-conjugated secondary antibody (1:500, Invitrogen) for 2.5 h at room temperature. Sections stained for GFP were additionally incubated in streptavidin-conjugated Alexa Fluor 488 (1:500, Invitrogen). After a final serial wash in TBS, the sections were mounted on slides to fully dry for 24 h and subsequently imaged using a Leica DM5500 fluorescent microscope.

#### Quantification

To determine the number of serotonergic and noradrenergic neurons in the graft, one series of longitudinal thoracic spinal cord sections immunostained for 5-HT, GFP, and DBH was examined under a fluorescent microscope. The lesion site was examined at 200× magnification and the number of 5-HT^+^ and DBH^+^ was counted separately. In each rat, the total number of each cell type was calculated and averaged in three sections, which contained the IML region, for statistical analysis. Under fluorescent microscopy, the distance of GFP^+^ axonal growth was measured using a calibrated eyepiece with a scale incorporated into the objective lens at 200× magnification. The longest distance of GFP^+^ axons from the caudal edge of the graft was then averaged across each section for statistical analysis.

### Statistical analysis

Sample sizes were determined based on our previously published work. Resting hemodynamic and CRD-induced AD data were separately assessed using a one-way ANOVA followed by Tukey's post hoc. For temporal changes in resting MAP and HR, values were assessed using a one-way repeated–measure (RM) ANOVA followed by Tukey's post hoc. As the data from spontaneous AD experiments were non-normally distributed, the nonparametric Kruskal–Wallis analysis was used followed by Dunn's post hoc. A paired *t* test was used for neural and hormonal regulation tests with vehicle and drug treatment, while an unpaired Student's *t* test was performed for histological data. A value of *p* < 0.05 was considered significant, and all data were expressed as mean ± standard error of mean (SEM). All statistical evaluations were conducted using the GraphPad Prism 9 software.

## Results

### Similar hemodynamic disorders occur after spinal cord crush or transection

**Figure 2.**
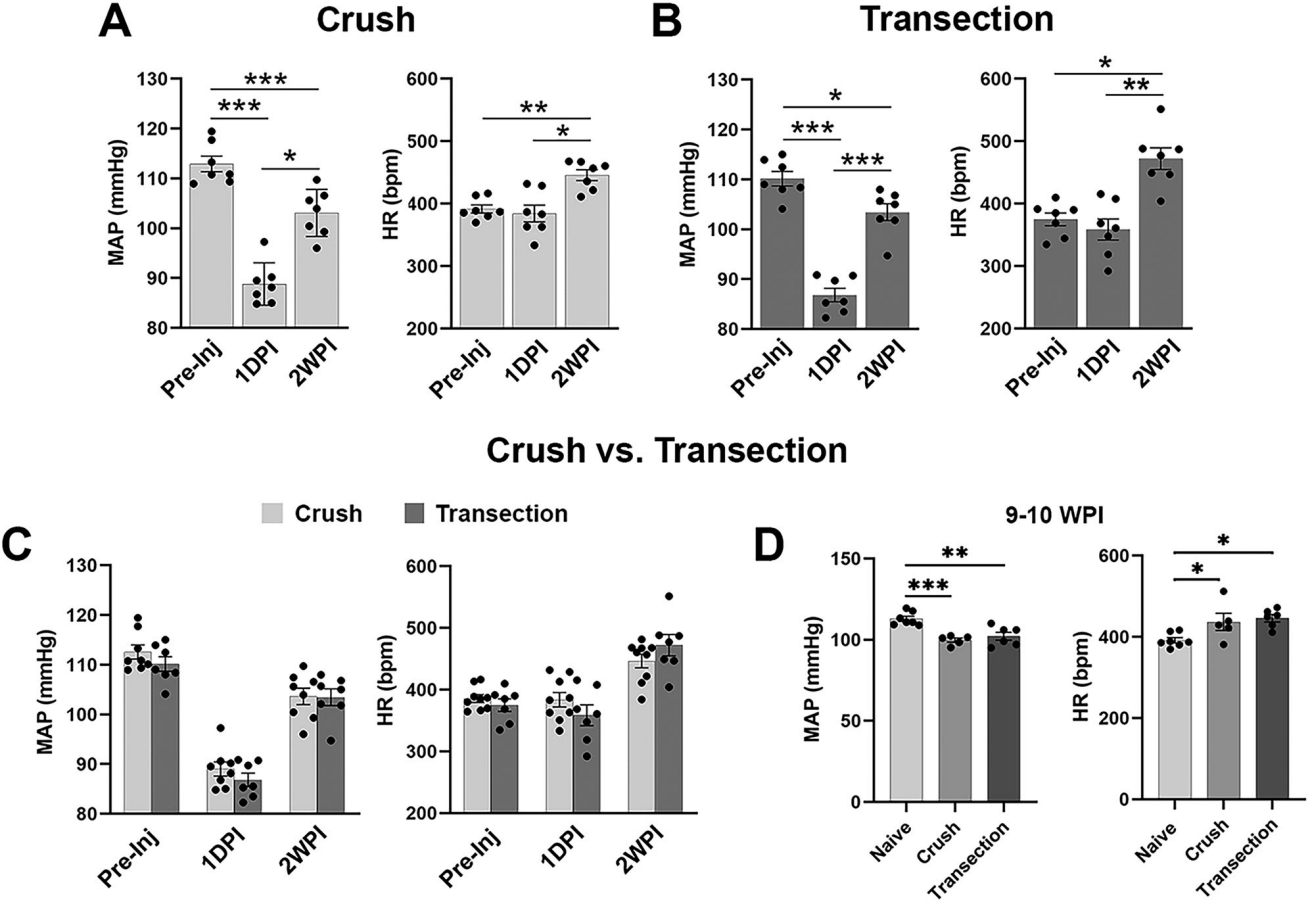
Comparison of temporal hemodynamic changes between rats with spinal cord crush and transection (*n* = 5–9 per group). ***A***, In rats with crushed SCI at the T4 level, MAP is significantly decreased at 1DPI and partially recovered at 2WPI. Resting HR is significantly higher at 2WPI compared with the preinjury state or 1DPI. ***B***, Similar hemodynamic changes occur in rats with spinal cord transection. ***C***, Statistical analysis suggests no overt differences in MAP or HR between crushed and transected animals at these acute or subacute time points (*p* > 0.05). ***D***, At 9–10WPI, similar reduced MAP and increased HR are observed in rats with crushed and transected SCI (*p < 0.05; **p < 0.01; ***p < 0.001).

Hemodynamic changes after crush SCI in the acute (1DPI) and subacute stages (2WPI) were first validated in comparison with a well-established spinal cord transection model. After T4 spinal cord crush, resting MAP significantly decreased 1DPI (one-way RM ANOVA, *p* < 0.001; Tukey's post hoc, *p* < 0.001) and later increased 2WPI but was still significantly less than preinjury MAP (*p* < 0.05). Meanwhile, resting HR remained unchanged 1DPI (one-way RM ANOVA, *p* < 0.01; Tukey's post hoc, *p* > 0.05) but significantly increased 2WPI (*p* < 0.05; Fig. 2*A*,*B*), indicating tachycardia. Notably, statistical analysis revealed that there were no differences in resting MAP and HR between the crush and transection models at these two time points (unpaired *t* test, all *p* > 0.05; Fig. 2*C*). To compare their hemodynamics at the chronic phase, we analyzed three experimental groups, including the naive, crush injury, and transection at the T4 spinal level. At 9–10 WPI, similar reduced MAP and increased HR were observed in rats with crushed and transected SCI in comparison with naive rats (one-way ANOVA followed by Tukey's post hoc; *p < 0.05; **p < 0.01; ***p < 0.001; Fig. 2*D*). The results suggest that hemodynamics is disordered similarly after T4 spinal cord crush and spinal transection in rats.

### NPC grafts improve cardiovascular performance

#### Blood pressure and HR at rest

**Figure 3.**
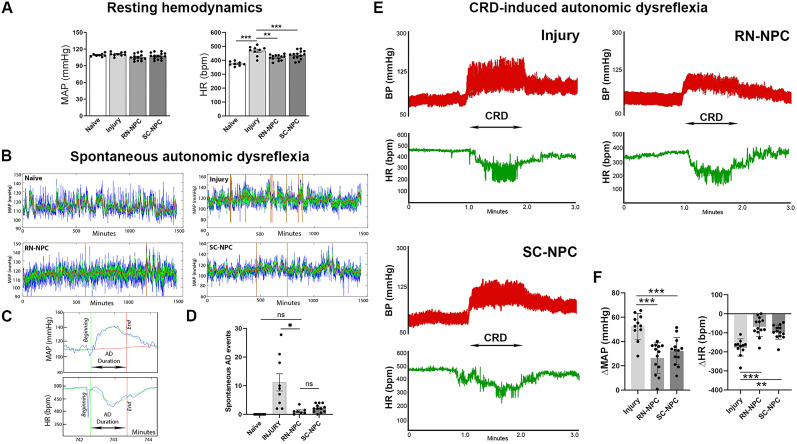
Transplanting NPCs into the injured spinal cord improves cardiovascular performance (*n* = 8–14 per group). ***A***, NPC grafts enhance resting cardiovascular function 10 weeks post-SCI. Resting HR in injury-only rats is increased and resting MAP does not change. Grafting with RN- or SC-NPCs significantly decreases resting HR but not to the level of naive controls. ***B–D***, Incidences of spontaneous AD decrease after NPC grafting. ***B***, A representative tracer of naturally occurring AD events over 24 h is depicted. ***C***, A vertical red line indicates an AD episode as detected using the established algorithm in MATLAB. A higher magnification of a detected event displays hypertension and concomitant bradycardia. ***D***, Statistical analysis reveals the number of spontaneously occurring AD events is significantly lower in RN-NPC–grafted rats compared with the injury alone. Notably, there is no significant (ns) difference between two grafted subjects. ***E***, ***F***, NPC transplantation alleviates CRD-induced AD severity. ***E***, Representative tracers illustrate AD episodes in each cohort. ***F***, Compared with injury alone, both RN- and SC-NPC–grafted rats have smaller changes in MAP and HR (*p < 0.05; **p < 0.01; ***p < 0.001).

Hemodynamic parameters, MAP and HR, at rest were recorded at 9–10 weeks after crush injury/grafting and compared between groups, including RN- or SC-NPC–grafted rats, injury alone, and naive controls. Statistical analysis revealed no significant differences in MAP between any of the groups (one-way ANOVA, *p* > 0.05). On the other hand, rats receiving injury alone displayed a higher HR than naive controls (one-way ANOVA, *p* < 0.0001; Tukey's LSD, *p* < 0.0001). Compared with injury controls, RN- or SC-NPC grafting significantly decreased HR (RN-NPC, *p* = 0.001; SC-NPC, *p* = 0.04). However, HR of both grafted groups was still higher than naive rats (RN-NPC, *p* < 0.01; SC-NPC, *p* < 0.0001; Fig. 3*A*). These data indicate that both RN- and SC-NPC grafts attenuate SCI-induced tachycardia, while the recovery is not back to the normal level.

#### Spontaneous AD events

The number of spontaneous AD events was examined over a 24 h period of 9 weeks after injury. As expected, no events were detected in naive rats, whereas those with injury alone experienced multiple AD episodes (Fig. 3*B*,*C*; Kruskal–Wallis, *p* < 0.0001). After RN-NPC grafting, the number of AD events was significantly lower than the injury alone (1.1 ± 1.1 vs 11.2 ± 9.0; Dunn's test, *p* < 0.05) and reached levels comparable to the naive (*p* > 0.05). The events in rats grafted with SC-NPCs showed a nonsignificant reduction (2.2 ± 1.4 vs 11.2 ± 9.0; Dunn's test, *p* = 0.23) compared with the injury alone. Notably, this parameter did not significantly reduce (Dunn's test, *p* = 1.0) between animals grafted with RN- or SC-NPCs (Fig. 3*D*). Overall, the results indicate that RN-NPC grafting tremendously decreases the emergence of spontaneous AD episodes.

#### CRD-induced AD

To further examine the severity of AD, we performed CRD to mimic bowel obstruction and induce dysautonomia in an experimentally controlled fashion in all SCI groups. Noxious CRD reliably elicited AD responses in all animals (Fig. 3*E*). However, the degree of MAP and HR change differed between groups. Compared with injury alone, rats grafted with RN-NPCs displayed significantly smaller changes in MAP (one-way ANOVA, *p* < 0.0001; Tukey's LSD, *p* < 0.001) and HR (one-way ANOVA, *p* < 0.001; Tukey's LSD, *p* < 0.001). SC-NPC–grafted rats also displayed a smaller increase in MAP and decrease in HR during CRD than injured controls (both *p* < 0.001; Fig. 3*F*). Thus, either RN- or SC-NPC grafts decrease the severity of CRD-induced AD.

### Unbalanced neuroendocrine regulation following SCI

**Figure 4.**
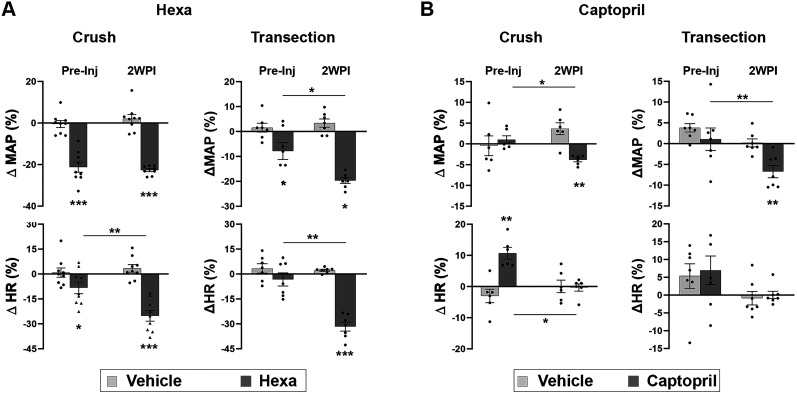
Comparison of neuroendocrine regulation of hemodynamics between spinal cord crushed and transected rats (*n* = 7/9 per group). ***A***, In rats with crush SCI, administration of the ganglionic blocker Hexa reduces resting MAP and HR either preinjury or 2WPI. Similar responses are seen when Hexa is administered to rats with spinal cord transection. ***B***, Delivery of the ACE inhibitor captopril has no significant effect on resting MAP in preinjury animals but induced a large decrease at 2WPI. Furthermore, the percentage decrease is markedly greater at 2WPI versus the preinjury state. Simultaneously, a significantly larger increase in HR is detected preinjury but not at 2WPI. This response is similar to that in rats with spinal cord transection (*p < 0.05; **p < 0.01; ***p < 0.001).

Previous studies reported reduced neural but increased hormonal regulation of blood pressure in rats with high-level spinal cord transection (Lujan et al., 2018). To examine if the crush model replicates this change, we first compared the indices between these two SCI rats. In crushed injured rats, intraperitoneal administration of Hexa significantly reduced resting MAP in both preinjury and 2WPI (paired *t* test; both *p* < 0.001). There was no difference in the percentage change in MAP after Hexa administration between the two postinjury points. Additionally, blocking ganglionic transmission induced bradycardia at both time points (preinjury, *p* < 0.05; 2WPI, *p* < 0.001). Hexa induced a greater bradycardic response at 2WPI than preinjury (*p* < 0.01; Fig. 4*A*), suggesting altered neural hemodynamic control, potentially due to increased cardiac sympathetic tone postinjury. In parallel, delivery of the ACE inhibitor captopril had a minimal effect on resting MAP in preinjury animals. In contrast, MAP decreased (*p* < 0.01) in response to the drug at 2WPI, suggesting that RAS reliance on vasoconstriction increases after SCI. At the same time, captopril increased resting HR preinjury (*p* < 0.01), while this effect was not seen at 2WPI (*p* > 0.05; Fig. 4*B*). This may be related to baroreflex dysfunction after SCI. When Hexa or captopril was administered to rats with spinal cord transection, similar responses were seen, which was decreased neural control with a concomitant increase in RAS regulation of blood pressure. Therefore, the T4-crush SCI model produces hemodynamic changes similar to those seen after complete spinal transection.

### Alterations of neuroendocrine regulation of blood pressure following chronic SCI and the effect of NPC grafts

Cross talk between autonomic and RAS systems is known. To isolate pure neural (sympathetic) modulation of blood pressure without hormonal influence, we administered captopril to block the RAS effects before delivering Hexa. The contribution was calculated as the decrease in MAP after the second drug relative to the value after the first drug.

**Figure 5.**
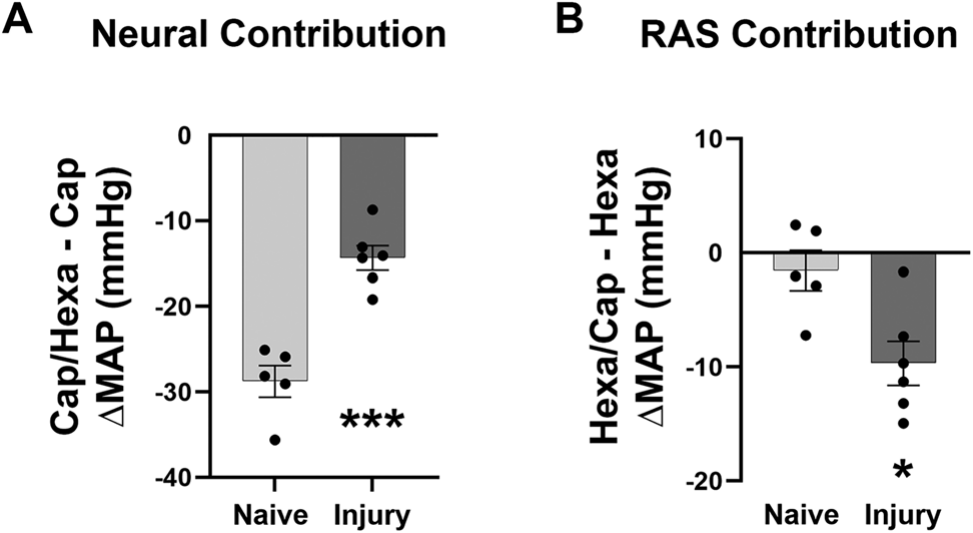
Chronic alterations in neuroendocrine regulation of blood pressure after SCI (*n* = 5–6 per group). Nine weeks after T4 crushed SCI, dual drug delivery was performed to test neuroendocrine regulation to compare with naive rats. ***A***, The hormonal effect is preblocked by captopril. Hexa induces a smaller change in MAP in SCI rats compared with naive controls. ***B***, The neural regulation is preblocked by Hexa. Captopril causes a greater change in MAP in SCI rats than naive controls (*p < 0.05; ***p < 0.001).

To determine the changes in neuroendocrine regulation of blood pressure at the chronic phase of SCI, we performed the dual drug administration 9 weeks after crushed SCI, and we compared the results with naive rats. After captopril injection to preblock RAS activity, Hexa induced a significantly smaller MAP reduction in SCI rats than naive controls (unpaired *t* test, ***p < 0.001; Fig. 5*A*). This indicates a reduced neural regulation of blood pressure after SCI. In contrast, when captopril was given after Hexa (to block neural regulation first), it caused a larger MAP reduction in SCI rats than in naive controls (*p < 0.05; Fig. 5*B*), indicating enhanced RAS-dependent blood pressure regulation post-SCI.

**Figure 6.**
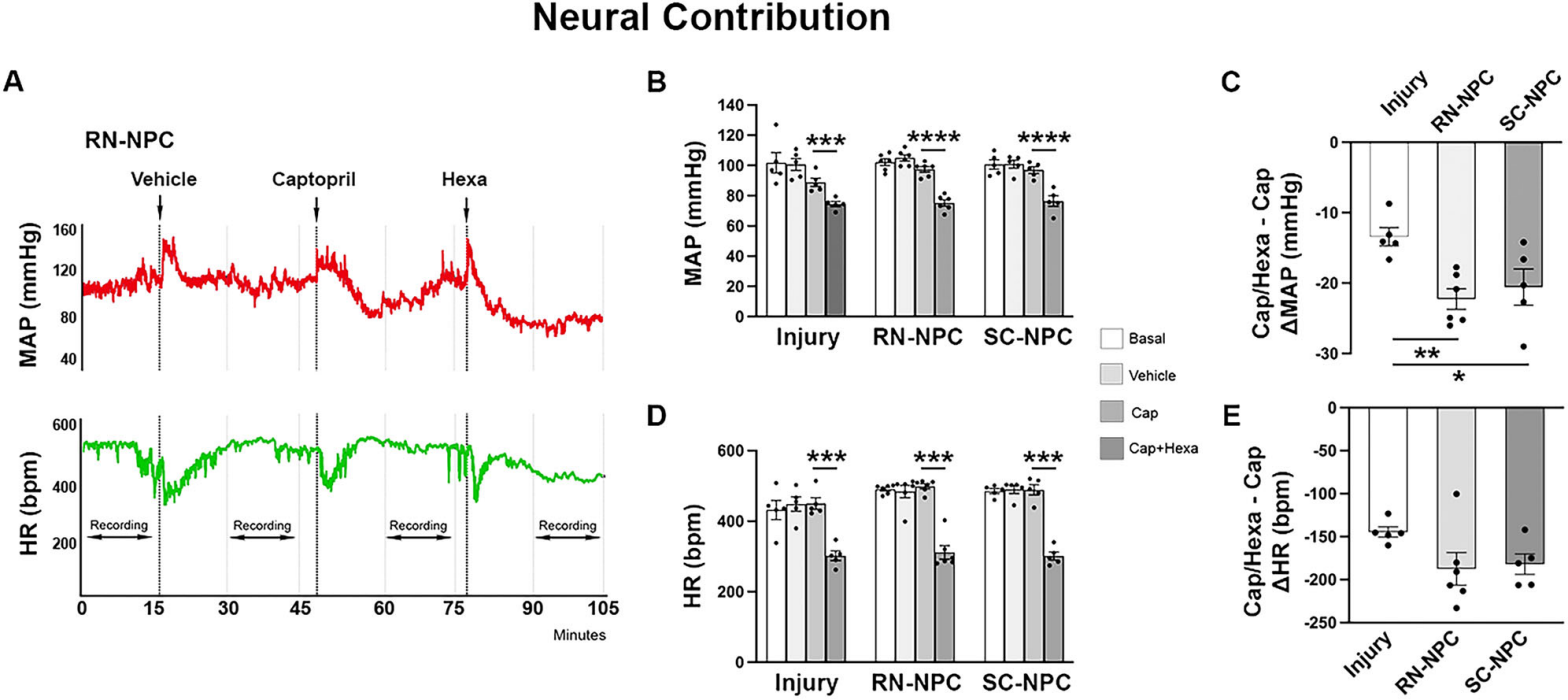
Neural regulation of hemodynamics augments after NPC transplantation (*n* = 5–6 per group). ***A***, A representative trace of resting MAP and HR after delivery of captopril/Hexa 9 weeks after RN-NPC grafting. ***B–E***, The hormonal effect is preblocked by captopril. ***B***, ***D***, The ensuing injection of Hexa causes a significant reduction of MAP and HR in all groups (***p < 0.001; ****p < 0.0001). ***C***, ***E***, Compared with injury controls, the extent of change in MAP is greater in both grafted groups. No significant difference in HR (*p < 0.05; **p < 0.01).

In grafting experiments, captopril alone significantly reduced MAP in injury controls in comparison with vehicle treatment (one-way RM ANOVA, *p* < 0.05; Dunnett's post hoc, *p* < 0.01) while slightly decreasing the parameter in both grafted groups (both *p* > 0.05). After the second drug Hexa was injected, MAP dropped significantly in all groups. Both NPC- and SC-grafted rats displayed a larger change in blood pressure (both *p* < 0.0001) than injury controls (Fig. 6). Compared with injury controls, the extent of change was greater in RN- (one-way ANOVA, *p* < 0.01; Tukey's post hoc, *p* < 0.01) or SC-NPC (*p* < 0.05)-grafted rats. Although Hexa significantly reduced HR in injury controls and grafted rats (all *p* < 0.001), the magnitude of reduction did not differ between groups. This reveals an increase in neural control of blood pressure after transplantation of NR- or SC-NPCs.

**Figure 7.**
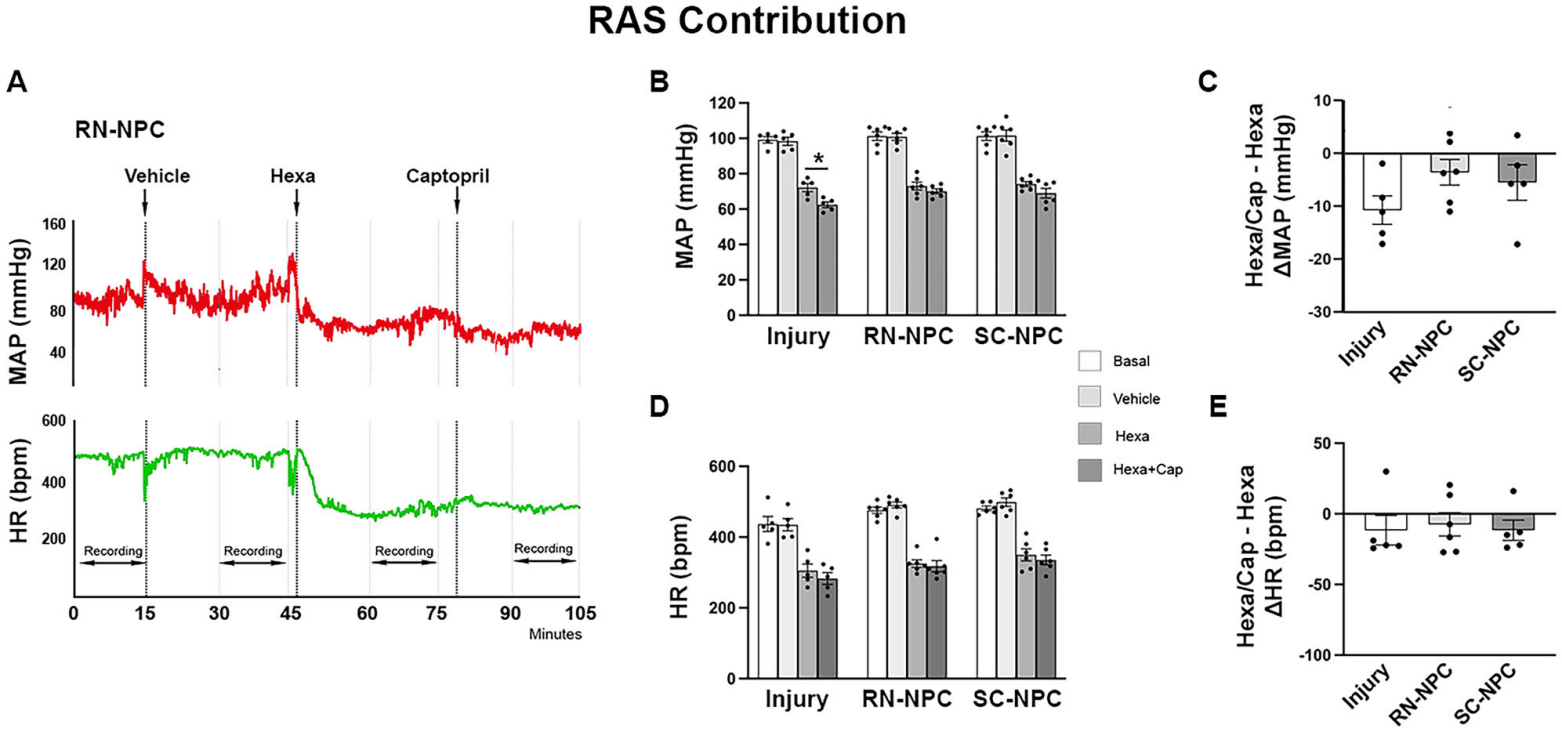
NPC grafting does not reduce elevated RAS regulation of hemodynamics (*n* = 5–6 per group). ***A***, A representative trace of resting MAP and HR after delivery of Hexa/captopril 9 weeks after RN-NPC grafting. ***B–E***, The neural regulation is preblocked by Hexa. Captopril decreases MAP in injury controls (*p < 0.05; ***B***). There is no statistical difference in both MAP and HR changes (***C***, ***E***) between injury controls and both grafted groups (all *p* > 0.05).

In parallel, Hexa was administered followed by captopril to examine pure hormonal regulation. Compared with the vehicle, Hexa alone caused a dramatic decrease in both MAP and HR (one-way RM ANOVA, *p* < 0.01; Dunnett's post hoc, all *p* < 0.001) in all three groups (Fig. 7). It was effective at decreasing MAP when the second drug captopril was delivered in injury controls (*p* < 0.05). However, the changes in MAP and HR with pure RAS regulation had no significant difference between the injury control with both grafted groups (Fig. 7*C*,*E*). These results demonstrate that NPC grafting from either the raphe or spinal cord restores neural regulation but does not alter SCI-induced RAS reliance of cardiovascular function.

### Spinal 5-HT_2A_ receptor blockade diminishes the recovery of resting HR

**Figure 8.**
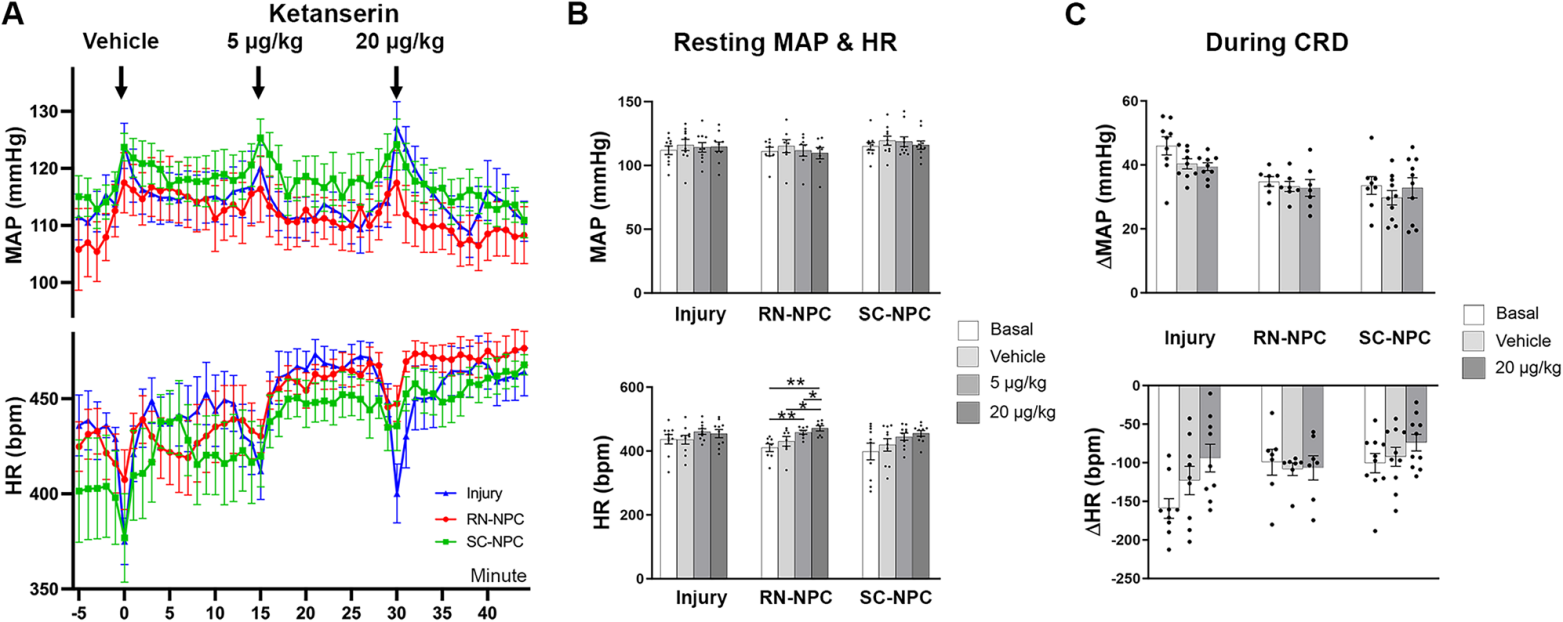
Administration of ketanserin diminishes restoration of resting HR (*n* = 8–10 per group). ***A***, ***B***, Nine weeks after SCI/grafting, the 5-HT_2A_ receptor antagonist ketanserin is administered intrathecally in serial dosages (5, 20 µg/kg). ***A***, Temporal analysis reveals no significant differences in MAP and HR at any time point compared with vehicle injection in RN-NPC–grafted rats. ***B***, The low and high doses increase resting HR in RN-NPC–grafted rats compared with the basal state, and the parameter value with the high dose was significantly greater than vehicle treatment, indicating the elimination of HR recovery established by grafts. ***C***, Ketanserin does not produce significant changes in MAP or HR during CRD-induced AD in all groups (*p < 0.05; **p < 0.01).

Spinal 5-HT_1A_ and 5-HT_2A_ are known central modulators of cardiovascular function (Ramage, 2001). To determine how graft-derived serotonin affects cardiovascular function, we intrathecally administered two 5-HT receptor blockers, ketanserin and WAY, during resting hemodynamic recordings and CRD-induced AD. For each treatment, resting MAP and HR were averaged over a 15 min period. Inhibition of spinal 5-HT_2A_ receptors with ketanserin did not affect MAP in any group, interestingly, administering a low (5 µg/kg) or high (20 µg/kg) concentration of ketanserin significantly increased resting HR in rats grafted with RN-NPCs compared with the basal (one-way RM ANOVA, *p* < 0.001; Dunnett's post hoc, both *p* < 0.01), and the value of the parameter with the high dose was significantly greater than vehicle treatment (*p* < 0.05). No such increase was seen with this dose in the SC-NPC group or injury controls (both *p* > 0.05; Fig. 8*A*,*B*).

Delivery of WAY to block spinal 5-HT_1A_ receptors did not elicit changes in resting MAP or HR in any group. Moreover, spinal 5-HT_1A_ (data now shown) or 5-HT_2A_ (Fig. 8*C*) receptor blockade did not alter CRD-induced AD severity in any group (all *p* > 0.05). Therefore, RN-NPC grafts modulate resting HR but not AD via 5HT_2A_ receptor signaling.

### Specific blockade of spinal α_1_-adrenoreceptors attenuates AD in NPC-grafted rats

**Figure 9.**
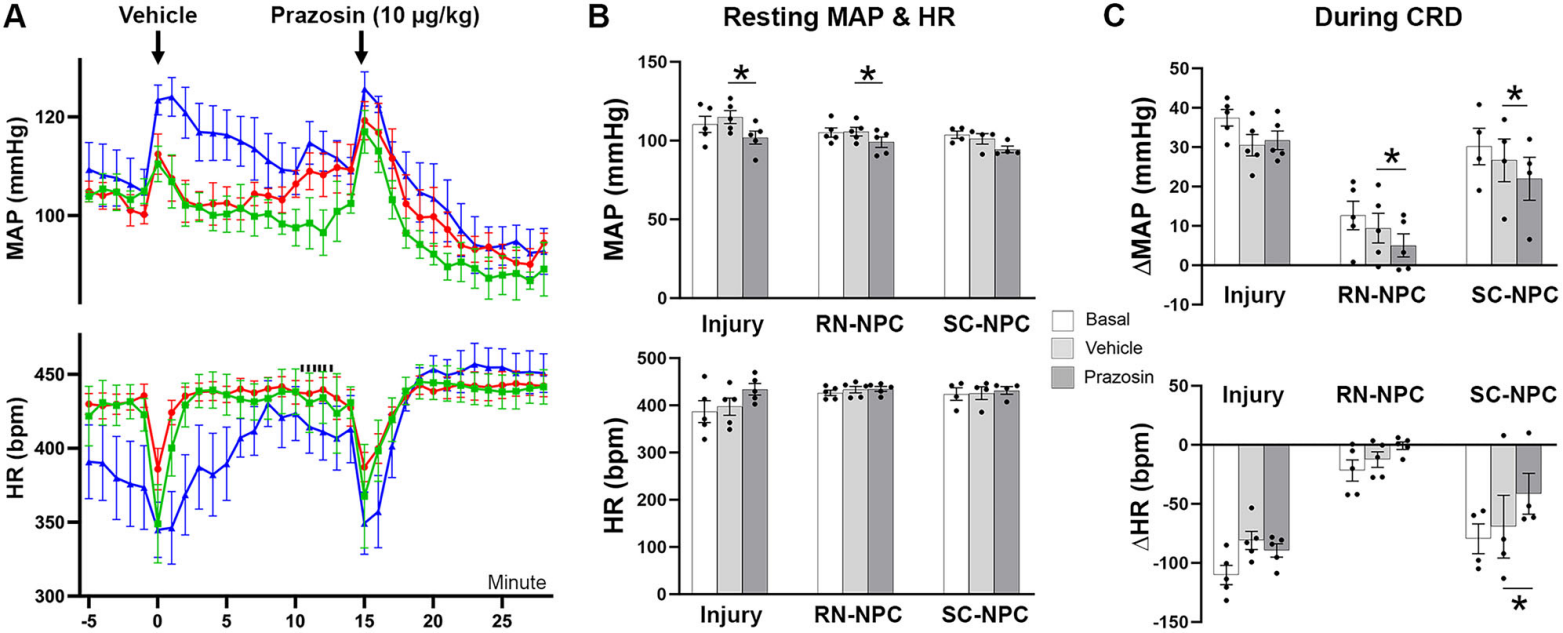
Administration of prazosin decreases resting blood pressure and graft-related dysreflexic responses (*n* = 4/5 per group). The α1 adrenoreceptor antagonist prazosin is administered intrathecally 9 weeks after SCI/grafting. ***A***, Temporal analysis reveals a significant decrease in MAP toward the end of the treatment recordings when compared with vehicle injections in both RN-NPC and injury-only groups. There is no interaction between time and treatment on HR among any groups. ***B***, Statistical analysis indicates that prazosin delivery significantly decreases resting MAP in RN-NPC–grafted and injury-only groups. A nonsignificant change is seen in the SC-NPC group. There is no effect on resting HR in any cohort. ***C***, Delivery of prazosin before CRD reduces the change of MAP in RN- or SC-NPC–grafted rats. However, this effect is not seen in the injury-only cohort (*p < 0.05).

We first examined whether administration of prazosin (1.0 µg/kg), an α_1_-adrenoreceptor antagonist, affected resting hemodynamics. Prazosin had no acute effect on MAP, which remained comparable to vehicle in all groups. However, over time, MAP did change significantly (one-way RM ANOVA, Dunnett's post hoc, all *p* > 0.05). RN-NPC–grafted animals showed a significant MAP reduction 8–12 min post-prazosin (8, 9, and 12 min, all *p* < 0.05; 10, 11 min, all *p* < 0.01). A similar decrease was observed in the injury-only animal cohort (11–13 min, all *p* < 0.05; Fig. 9*A*).

Central prazosin administration reduced resting MAP in RN- or SC-NPC–grafted rats versus vehicle (one-way RM ANOVA, *p* < 0.05; Dunnett's post hoc, RN-NPC *p* < 0.05; SC-NPC *p* = 0.16). Importantly, the drug also lowered MAP in injury controls (*p* < 0.05). No effect was seen on resting HR in all groups (Fig. 9*B*). This suggests that this noradrenergic/adrenergic mechanism is still maintaining blood pressure after SCI, regardless of the presence of NPC grafts.

During CRD-induced AD, prazosin significantly attenuated episodic hypertension in both RN- (*p* < 0.05) and SC-NPC (*p* < 0.01)-grafted groups. The drug significantly reduced HR change in SC-NPC–grafted rats (*p* < 0.05) and showed a trend in RN-NPC–grafted rats (*p* = 0.08). This was not detected in SCI-only animals (both *p* > 0.05; Fig. 9*C*). These data indicate that the α_1_-adrenoreceptors contribute to AD in NPC-grafted rats.

### Grafted cell survival, integration, and projection

**Figure 10.**
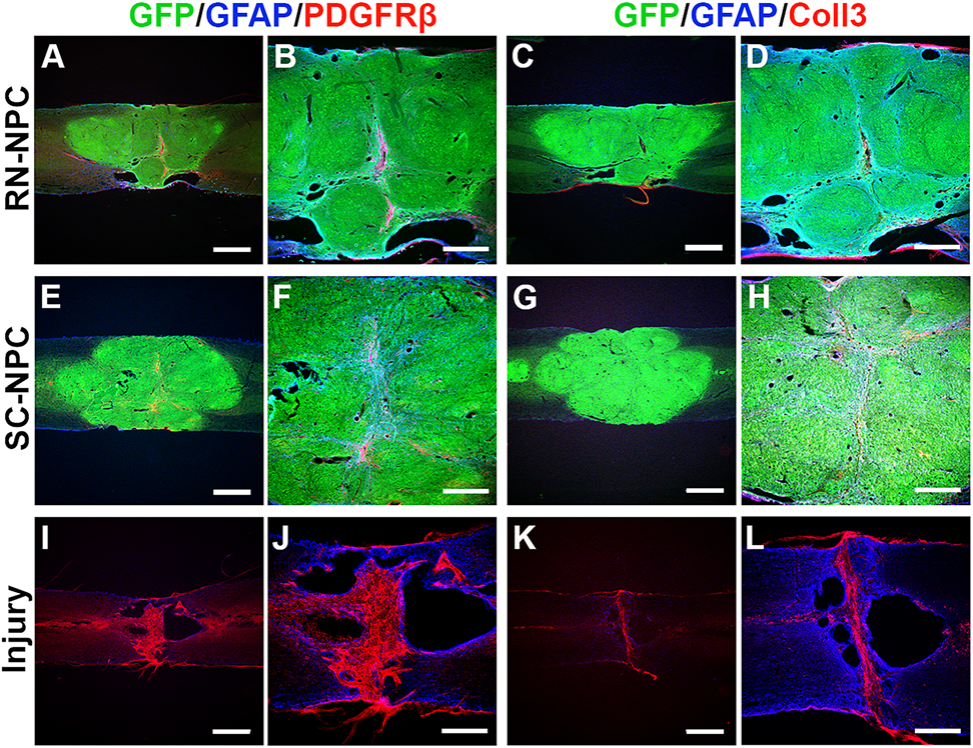
Grafted cells survive and integrate with the host tissue after transplantation (*n* = 5/6 per group). Both transplanted RN- and SC-NPCs survive and integrate with the host spinal cord tissue. Very few PDGFR-β^+^–labeled fibroblasts and GFAP-labeled astrocytes are present at the lesion center (***A***, ***B***, ***E***, ***F***). Similarly, there is little expression of Collagen 3 (Coll 3), another marker for fibrotic scarring, in the graft epicenter (***C***, ***D***, ***G***, ***H***). In injury-only spinal cords, a dense labeling of both (***I***, ***J***) PDGFR-β^+^ cells and (***K***, ***L***) Coll 3 spans across the entire lesion. Scale bars: ***A***, ***C***, ***E***, ***G***, ***I***, ***K***, 1 mm; ***B***, ***D***, ***F***, ***H***, ***J***, ***L***, 500 µm.

**Figure 11.**
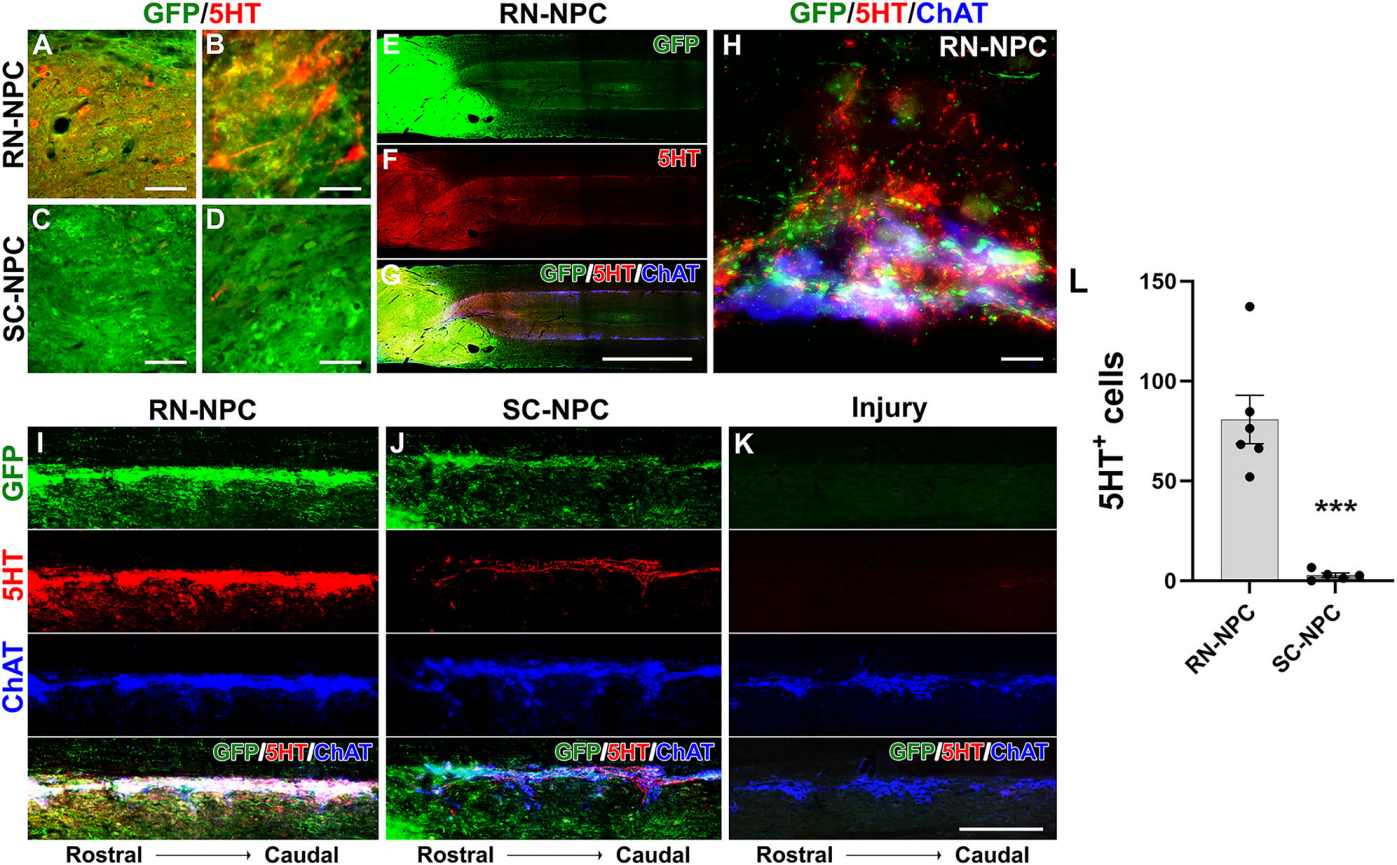
RN-NPC graft-derived serotonergic neurons extend long-distance axons along caudal autonomic regions (*n*= 5/6 per group). ***A–D***, Nine weeks posttransplantation, immunostaining reveals many serotonergic cells (5-HT^+^) in RN-NPC but very few in SC-NPC grafts. ***E–G***, In a representative RN-NPC–grafted cord, transplant-derived serotonergic axons project along bilateral IML cell column for segments, innervating SPNs labeled with choline acetyltransferase (ChAT). ***H***, A higher magnification shows the close juxtaposition of these axon terminals to SPNs. ***I***, ***J***, RN-NPCs derive dense 5-HT^+^ axons along the IML below the lesion, while SC-NPC derives few and none in rats with injury only. ***L***, Statistical analysis suggests that there is a larger population of serotonergic (unpaired *t* test, ***p < 0.001) neurons in RN-NPC than SC-NPC grafts. Scale bars: ***A***, ***C***, 100 µm; ***B–D***, 50 µm; ***G***, 2 mm; ***H***, 33 µm; ***K***, 200 µm.

The integration of transplants and host spinal tissue was qualitatively examined through immunohistochemical analysis. In the spinal cord with crush injury alone, there was notable fibrotic scarring, denoted by PDGFRβ and Collagen III, gliosis identified by GFAP staining, as well as cavitation, in the lesion site. In NPC transplanted cords, triple immunostaining demonstrated that GFP-labeled grafted RN- or SC-NPCs survived well at the lesion site. The grafts bridged the rostral and caudal stumps of the spinal cord and fused with the host tissue. Fibroglial scarring and cavitation were rare in NPC-grafted cords. Thus, both RN- and SC-NPC cells survived and integrated with host tissue across the lesion (Fig. 10). RN-NPC grafts contained significantly more 5-HT^+^ serotonergic neurons than SC-NPC grafts (Fig. 11*A*–*D*). Furthermore, grafted NPCs extended long-distance axons to the caudal spinal cord. In RN-NPC–grafted cords, 5-HT^+^ axons innervated the caudal autonomic region, the IML cell column, and the dorsal gray commissure. These projections were colocalized with GFP, suggesting a graft-derived but not host supraspinal origin (Fig. 10*E*–*I*). Under high magnification, these 5-HT^+^ fibers were closely juxtaposed to SPNs in the IML region (Fig. 11*H*). In contrast, SC-NPC grafts derived a few 5-HT^+^ axons extending along the region (Fig. 11*J*). No immunolabeled axons were detected in the caudal IML region in injury controls (Fig. 11*K*). Statistical analysis indicated that the number of 5-HT^+^ cells was significantly higher in RN-NPC grafts (80.8 ± 12.1) than in SC-NPC grafts (2.7 ± 1.4; unpaired *t* test, *p* < 0.001; Fig. 11*L*).

**Figure 12.**
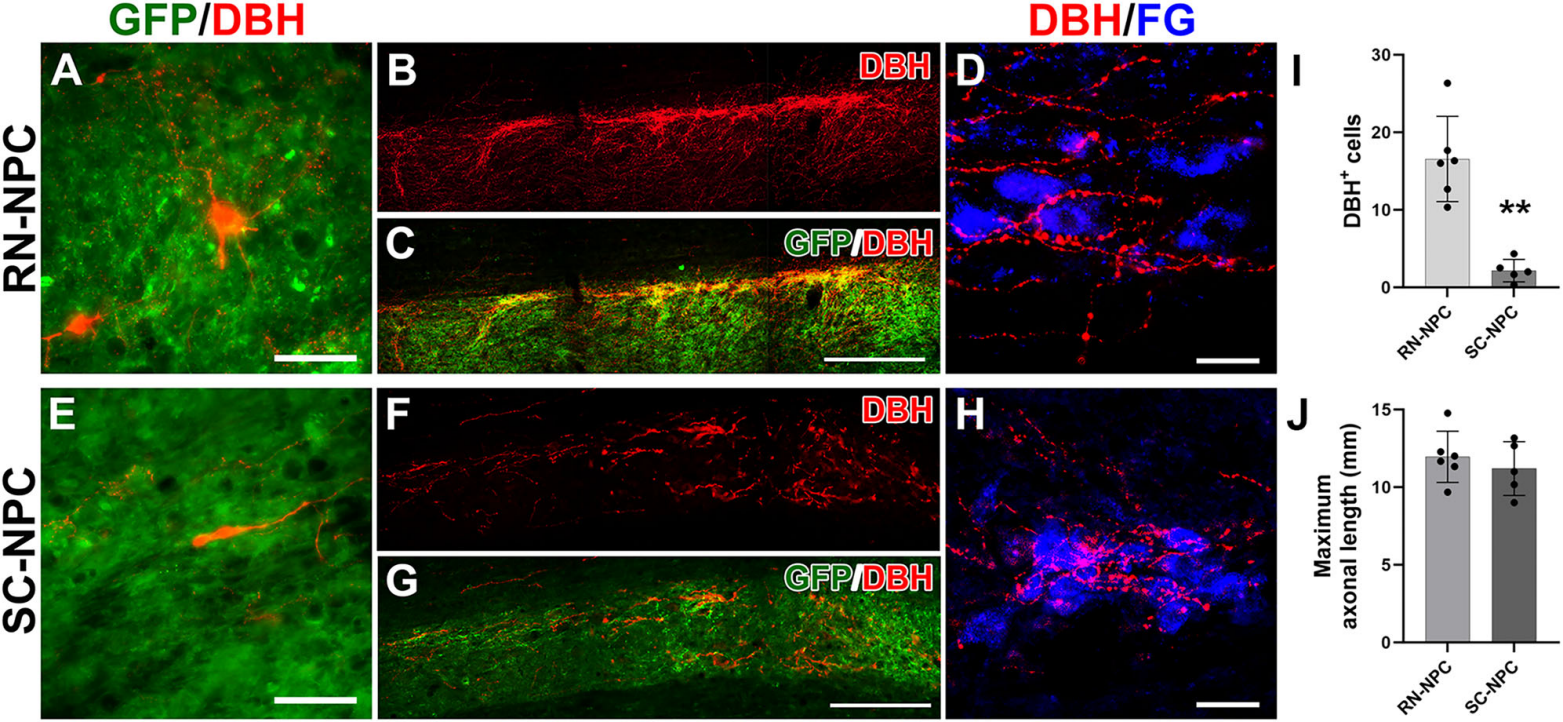
Graft-derived noradrenergic neurons extend axons along autonomic regions (*n* = 5/6 per group). Nine weeks posttransplantation, immunostaining reveals some noradrenergic cells (DBH^+^) in NPC grafts (***A***, ***E***). RN-NPC grafts project DBH^+^ axons caudally along the IML column in the cord (***B***, ***C***), while SC-NPC grafts extend a few (***F***, ***G***). A higher magnification indicates DBH^+^ axon terminals intermingled with FG-labeled SPNs. Statistical analysis shows (***I***) more DBH^+^ cells in RN- than SC-NPC grafts (unpaired *t* test, **p < 0.01). ***J***, The distance of GFP^+^ axonal elongation is not different between RN- and SC-NPC grafts. Scale bars: ***A***, ***E***, 50 µm; ***C***, ***G***, 200 µm; ***D***, ***H***, 33 µm.

**Figure 13.**
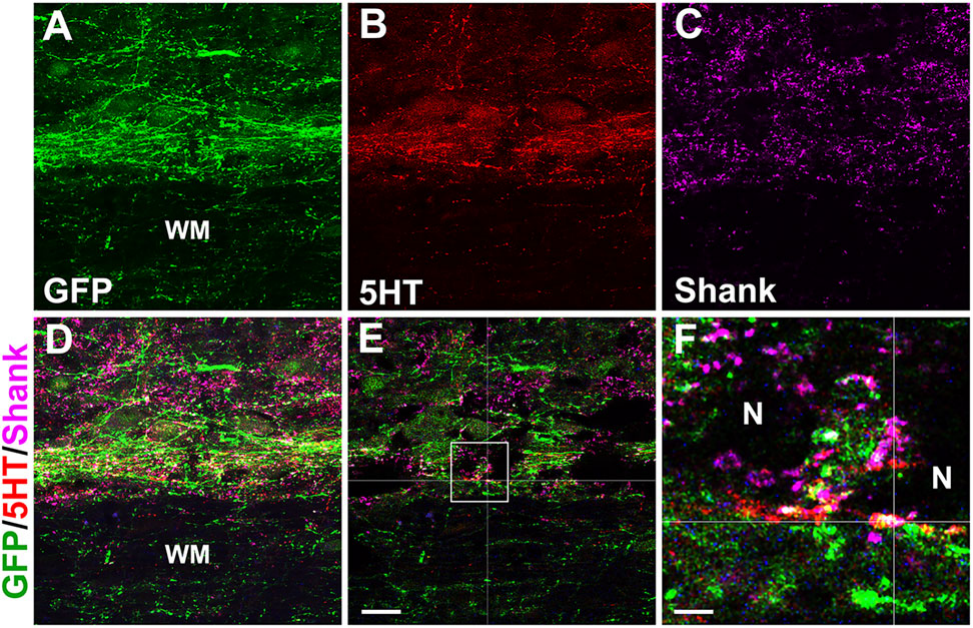
Graft-derived serotonergic axons form synapses with SPNs. Under confocal microscopy, immunostaining demonstrates that RN-NPC graft–derived 5-HT^+^ axon terminals overlapped with postsynaptic marker Shank, identified around SPN cell bodies, in the IML caudal to the injury/graft. ***E*** is a scanned single layer, and the boxed region is ***F***. Cross lines represent a triple-labeled profile (WM, white matter; N, neuron). Scale bars: ***E***, 20 µm; ***F***, 5 µm.

Though we enriched the serotonergic population by dissecting the raphe region, other types of neural cells are located in close proximity to this area, such as noradrenergic ones (Bernstein-Goral and Bohn, 1988; Hou et al., 2020), which could have been included in the graft. Indeed, a small portion of noradrenergic/adrenergic neurons expressing DBH were detected within NPC grafts (Fig. 12*A*,*E*). DBH^+^ axonal projections were observed in the autonomic regions caudal to the lesion/graft (Fig. 12*B*–*D*,*F*–*H*). Quantitative analysis demonstrated that there were more DBH^+^ cells in the RN-NPC (16.6 ± 2.2) than the SC-NPC transplant (2.1 ± 0.8; unpaired *t* test, *p* < 0.01; Fig. 12*I*). There was no difference in the maximal length of GFP^+^ axons between groups (RN-NPC 11.96 ± 0.67 vs SC-NPC 11.25 ± 1.00 mm; unpaired *t* test, *p* > 0.05; Fig. 12*J*). The length pattern of 5-HT^+^ or DBH^+^ fibers was consistent to GFP^+^ axons in both grafted groups. In addition, confocal microscopy analysis demonstrated that some terminals of graft-derived axon projections overlapped with postsynaptic marker Shank, identified around SPN cell bodies, in the IML caudal to the injury (Fig. 13). This indicates the likelihood of established communications between grafted neurons and the host sympathetic system. Collectively, RN-NPC grafts facilitate the reinnervation of monoaminergic projections onto autonomic regions of the caudal spinal cord, consistent with previous reports (Hou et al., 2013b, 2020).

## Discussion

Embryonic neuron grafts integrate robustly with the host tissue when transplanted into the lesion of a crushed spinal cord. RN- or SC-NPC implants decrease SCI-induced tachycardia and attenuate the frequency and severity of AD. Following high-level injury, neural regulation of blood pressure reduces, while RAS reliance increases in the chronic phase. NPC grafts, whether derived from brainstem or spinal cord tissues, enhance neural control of basal hemodynamics without changing RAS hyperactivity. Specifically, graft-derived intraspinal serotonin transmission is involved in the restoration of hemodynamics via 5-HT_2A_ receptors but not dysreflexic responses after grafting serotonergic neurons. Catecholaminergic signaling continues to maintain blood pressure through activation of the α_1_-adrenoreceptor after SCI.

As most peripheral vasculatures are innervated by sympathetic but not parasympathetic projections, neural regulation reflects the sympathetic drive on blood pressure. Following high-level SCI, supraspinal regulation of sympathetic input to the vasculature is largely lost. This dysregulation typically causes an acute drop in resting blood pressure. However, partial recovery of vasoconstrictor tone occurs over time, elevating basal blood pressure to a new set point below the naive level (West et al., 2015). We confirmed these hemodynamic changes in our crush injury model. It was previously reported that there is an increased reliance on the RAS for hemodynamic regulation at the chronic stage (Lujan et al., 2018), which is likely a compensatory mechanism to augment low blood pressure via bodily self-adjustment. Here, when we administered a ganglionic blocker Hexa or an ACE blocker captopril, it confirmed this out of balance in neuroendocrine regulation of hemodynamics in rats with SCI. We next sought to identify the possible changes in the balance between neural and hormonal regulation of cardiovascular function after NPC grafting.

Angiotensin II potentiates sympathetic vasomotor output. Previous studies reported that Ang II raises arterial blood pressure via central and peripheral mechanisms. Centrally, this vasoconstricting hormone activates central vasomotor regions, such as the nucleus tract solitarius and the rostral ventrolateral medulla (Wang et al., 2007; Allen et al., 2009). Ang II also directly excites SPNs via angiotensin-type 1 receptors (AT_1_R; Yashpal et al., 1989; Chao et al., 2013). Peripherally, this hormone maintains blood pressure through vascular AT_1_R activation and renal sodium retention. To isolate the pure role of each factor, we delivered the two drugs in a combinatorial method by eliminating interaction between these two components. Compared with uninjured animals, rats with high-level SCI displayed reduced neural control and an increased reliance on the RAS for blood pressure modulation in the chronic phase. To determine sympathetic drive independent of Ang II influence, we first blocked RAS activity by injection of captopril. Subsequent Hexa administration produced more robust effects on blood pressure, calculated as ΔMAP, in the grafted animals compared with injury controls. This suggests that both grafted NPCs improved neural regulation of blood pressure. Inversely, the compounded application of Hexa first to preblock neural component and then followed by captopril injection showed no significant difference in ΔMAP among groups, indicating the persistence of aberrantly escalated RAS reliance regardless of cell transplantation.

RN-NPC grafts contained an enriched population of serotonergic neurons. They likely released serotonin to the spinal cord after supraspinal transport was interrupted. Their projections extended long distances toward the caudal spinal cord and innervated SPNs within the IML. This suggests that RN-NPC grafts may reintroduce serotonergic regulation of sympathetic output. The intraspinal mechanisms underlying this phenomenon, however, remain largely elusive. Previously, RN-NPC–mediated basal cardiovascular restoration was found to be mediated in part through serotonergic mechanisms. Systemic ketanserin administration blunted blood pressure recovery exclusively in RN-NPC–grafted rats. This suggests grafted serotonergic neurons are involved in restoring a normotensive state through the activation of central 5-HT_2A_ receptors (Hou et al., 2020). Here, the results revealed that central delivery of high-dose ketanserin eliminated graft-derived restoration of resting HR. It was possible that blocking 5-HT_2A_ first decreased blood pressure, which in turn triggered baroreceptive machinery to increase HR for blood pressure maintenance. Regardless, these results suggest the involvement of RN-NPC graft–derived serotonergic neurons in cardiovascular improvement via intraspinal 5-HT_2A_ mechanisms. Given ketanserin's 2–5 h half-life in rats (Michiels et al., 1988), effects of the high dose (administered 15 min postinitial dose) may reflect pharmacokinetic accumulation. Blockade of 5-HT_1A_ receptors with WAY100635 did not alter resting hemodynamics, excluding their role in functional restoration.

Both RN- and SC-NPC grafts reduced CRD-induced changes in MAP and HR. Our previous transsynaptic tracing data (Hou et al., 2020) suggest that graft-derived mechanisms may inhibit exaggerated spinal reflexes through supraspinal relay and spinal interneurons connecting to SPNs. It was recently reported that subcutaneous administration of ketanserin depressed CRD responses in both grafted and injury-control rats, suggesting a peripheral mediation of AD suppression. Since the drug entry route might act on the vasculature, it cannot conclude the role of graft-derived serotonergic activity in diminishing AD severity (Hou et al., 2020). To circumvent this limitation, we administered ketanserin and WAY100635 intrathecal prior to CRD induction. Neither drug altered CRD-induced AD severity. This indicates that, while serotonergic pathways regulate resting sympathetic tone and reflex in the intact and SCI state (Danzebrink and Gebhart, 1991; Ramage, 2001; Cormier et al., 2010), graft-derived serotonergic neurons likely do not mediate AD attenuation.

Beyond serotonergic neurons, RN-NPC grafts contained other brainstem neuron populations, such as catecholaminergic subtypes immunolabeled with DBH. These nonserotonergic neurons may also contribute to cardiovascular improvement. Norepinephrine/epinephrine has two sources in the body, the central/peripheral neurons and the adrenal gland (Goldstein et al., 2003). Central delivery of the α1 antagonist prazosin reduced MAP in either grafted or injury-only groups. This indicates that catecholaminergic mechanisms maintain hemodynamics after SCI. Since supraspinal catecholaminergic pathways are interrupted after SCI, with no descending neurotransmitter transportation to the lower spinal cord (Hou et al., 2013b), the effect could be mediated through circulating catecholamine. Surprisingly, prazosin administration before CRD reduced MAP response in both RN- and SC-NPC–grafted animals, but not injury-only controls. This rapid reflex modulation likely reflects inhibition of transplants or reconstituted supraspinal pathways. Though SC-NPC grafts nonsignificantly reduced spontaneous AD events, the reduction was over 50% compared with injury-only controls, and it was not significantly different from RN-NPC grafts and naive state. Therefore, grafted NPCs have a clear therapeutic effect to improve spontaneous AD response.

Following SCI, resting hemodynamic disorders manifest as low blood pressure, tachycardia, or both in rats, depending on injury severity and level (Furlan and Fehlings, 2008). Our crush injury model produced tachycardia. A sustained normal level of blood pressure may reflect baroreflex compensation (Swenne, 2013). It has been shown that severing the dura during SCI exacerbates proinflammatory responses and leads to further cell death and secondary damage (Fernandez and Pallini, 1985; Song et al., 2014; Anjum et al., 2020). As the crush injury preserved the dura, this caused less scarring and cavitations versus transection injury (Hou et al., 2018; Trueblood et al., 2019). Nevertheless, cardiovascular outcomes are similar between crush and transection models, validating crush models for experimental therapeutic research. Humans with a midthoracic SCI often present with tachycardia at the chronic stage (Jacobs et al., 2002). This has also been displayed in animal models with a transection at the level of the upper thoracic spinal cord and is thought to be a result of increased arborization of sympathetic pre- and postganglionic neurons projecting to the heart. Following SCI, nerve growth factor is released from the cardiac tissue and transported back to post- and preganglionic neurons (Hassankhani et al., 1995; Ieda et al., 2004; Lujan et al., 2010), which has been heavily implicated in sympathetic hyperinnervation of the heart. In intravenous drug delivery, researchers often use opposite drugs to confirm the experimental effects. However, it is difficult to perform this manipulation in other delivery methods, such as intraperitoneal and intrathecal, due to slow kick-off response or very small dose administration. Thus, a shortage is that we did not implement an agonist delivery after the blockade to further verify the efficacy.

Although the chronic validation test showed hypotension and tachycardia after spinal cord crush, rats with crushed SCI alone in the grafting experiment exhibited tachycardia without hypotension. This discrepancy may reflect the variation of the lesion level in the spinal cord. The SPNs regulating cardiac activity reside at the T1–4 spinal level. A slightly higher injury level could damage heart-related SPNs through second injury of expansion, potentially decreasing stroke volume and triggering compensatory tachycardia via cardiac vagal withdrawal while maintaining normal blood pressure.

In summary, transplantation of embryonic NPCs enhances neural control of vascular tone, promoting cardiovascular recovery after SCI. Graft-derived serotonin signaling restores basal hemodynamics but does not attenuate AD. Catecholaminergic mechanisms continue to maintain blood pressure after SCI. Ultimately, the results provide new insight into the mechanistic basis of cell therapy for SCI-induced autonomic dysfunction. The future direction will point toward how to reduce excessive RAS dependence to optimize neuroendocrine homeostasis for cardiovascular recovery.

## Synthesis

Reviewing Editor: Viji Santhakumar, University of California Riverside Decisions are customarily a result of the Reviewing Editor and the peer reviewers coming together and discussing their recommendations until a consensus is reached. When revisions are invited, a fact-based synthesis statement explaining their decision and outlining what is needed to prepare a revision will be listed below. The following reviewer(s) agreed to reveal their identity: Samir Patel, Christopher West.

This manuscript presents an extensive study on NPC transplantation to recover neuroendocrine regulation of cardiovascular function in experimental spinal cord injury. The results are interesting and contribute to the understanding of transplantation-mediated recovery mechanisms. The manuscript is generally well written and follows a series of other studies that have investigated the usefulness of NPC transplantation to recover serotonergic control of CV function after SCI.

1. The whole first section attempts to validate the T4 crush injury against a transection injury, presumably to "show" that the crush injury produces a similar phenotype to that of a transection injury and is therefore a 'valid' model of CV dysfunction. Yet, the validation period only lasted 2 weeks, and not the 10 weeks that the rest of the study used. Can you please rationalize the choice of using an earlier time-point. Indeed, we know that these crush and contusion models have a natural recovery of CV function with time post-SCI. Indeed, data in Figure 2 show no reduction in MAP in crush injury-only group 10 weeks after injury.

2. Relatedly, the methodology indicates that a crush injury was induced using forceps, but additional clarification is necessary regarding the visual criteria used to ensure the injury was a complete crush. Providing specific parameters would enhance consistency across subjects. Additionally, whether exclusion criteria were established to address variability in injury severity needs to be indicated. Clearly defining these criteria would enhance study rigor.

3. There appears to be limited dysfunction in resting blood pressure 10 weeks after injury. The tachycardia could be a consequence of injury to SPNs projections to the heart - leading to decrease in stroke volume with a normal MAP, then cardiac output would also likely (though not measured) be normal. Consequently, the only way the rats can reach a normal cardiac output is through increasing HR by vagal withdrawal. This possibility needs to be considered and discussed. It would also be useful to quantify SPNs in T2-T6 segments in the groups.

4. Since previous publications using the same cells in the same injury model have shown recovery of cardiovascular function and reduced AD severity post-SCI, the novelty of the study hinges on pharmacology used to try and further interrogate how these cells reduce the severity of AD. This should be emphasized in the discussion.

5. The study lacks details on how graft-host integration was assessed. Please include information on the methods used to verify graft survival and functional integration.

6. Please detail how the raphe was identified and isolated in an E13.5 rat embryo. The data show that other cell types (i.e., DBH+) cells are also present in these grafts. How were the graft constituents / cell types confirmed - in the absence of culturing and restricting the fate, many cell types may be present in the graft.

7. Example traces provided in Figure 4 and 5 suggest that all drugs tested elicited 'spike' in BP at the time of infusion. Please indicate the volume of solution being injected for each of the drugs. The challenge is that high volume injections can lead to volume loading the heart prior to examination of the drug response, which will alter the baroreflex.

8. Please indicate if and how the efficacy of pharmacological blockade was confirmed. Was an agonist delivered after the blockade to ensure complete pharmacological blockade as intended.

9. There is a discrepancy in the timing of telemetric probe implantation across groups. In the transplantation group, probes were implanted 7-8 weeks after surgery, whereas in the validation group, implantation occurred one week before spinal cord injury. Justification for this difference would provide clarity on the experimental design.

10. There are many sections in the discussion that do not line up with the experimental data provided:

a. Opening section of discussion (p.29) it is stated that neuronal regulation of blood pressure is reduced. Based on what data? You have no difference in resting BP across all SCI group and HEX administration reduced BP by the same amount across all control or transplant groups (Figure 5).

b. It is suggested that that intraspinal serotonin transmission is involved in the restoration of hemodynamics - however, intrathecal administration of Ketanserin produced no change in BP in any group at rest or during CRD.

c. It is suggested that catecholaminergic mechanisms still maintain BP through activation of alpha-1 receptor. However, the alpha-1 receptor is mostly important in regulating PVN projections to the SPNS, not the RVLM projections (which synapse onto NMDA and/or mGluRs on SPNs). In the absence of stimulation and recording from the RVLM it is difficult to ascertain that these are catecholaminergic projections.

d. First main paragraph of the discussion - based on data presented the crush model presented here does not fully reproduce the reduction in resting BP seen clinically and in transection rodent studies. Data show nono reduction in MAP at 10 weeks post-SCI (only at 2 weeks when recovery is still ongoing). Moreover, HEX (Before Captopril) leads to a drop in BP by the same amount in all groups, implying 'normal' sympathetic regulation of BP in the model.

e. Please clarify the Captopril/Hexa arguments in the discussion. It appears that the only difference observed is that delivery of Captopril after HEX leads to a greater drop in MAP in the SCI-control group and not in the transplanted group. This would imply only that in the presence of a completely non-functioning SNS that SCI (non-transplanted) animals rely on angiotensin II to maintain their BP, whereas grafted animals do not - although this result does not appear to come out when looking at delta BP. If this is the case, the functional significance of this is unclear as the animals recover their SNS control over BP (as shown by the HEX-first blockade data).

f. The suggestions that data show that graft-derived serotonergic neurons are involved in cardiovascular improvement after RN-NPC transplantation through intraspinal 5HT2A mechanisms (page 32) needs additional clarification and validation. Figure 6 shows that i.t. administration of Ketanserin had no impact on resting BP or the BP response to CRD.

g. Page 32 - DBH+ neurons from the RVLM do not typically synapse on alpha-1 receptors on SPNs. Thus, this whole section to be overly speculative.

11. The phrase "reduced hormonal reliance" in the abstract is ambiguous and needs to be revised.
